# Comparative Genomics of Different Lifestyle Fungi in Helotiales (Leotiomycetes) Reveals Temperature and Ecosystem Adaptations

**DOI:** 10.3390/jof10120869

**Published:** 2024-12-14

**Authors:** Daniel Vasconcelos Rissi, Maham Ijaz, Christiane Baschien

**Affiliations:** Leibniz Institute DSMZ, German Collection of Microorganisms and Cell Cultures, 38124 Braunschweig, Germany; daniel.rissi@dsmz.de (D.V.R.); maham.ijaz@dsmz.de (M.I.)

**Keywords:** Helotiales, temperature adaptation, fungal lifestyles, genomic adaptations

## Abstract

Helotiales, a diverse fungal order within Leotiomycetes (Ascomycota), comprises over 6000 species occupying varied ecological niches, from plant pathogens to saprobes and symbionts. Despite their importance, their genetic adaptations to temperature and environmental conditions are understudied. This study investigates temperature adaptations in infection genes and substrate degradation genes through a comparative genomics analysis of 129 Helotiales species, using the newly sequenced genomes of *Gyoerffyella rotula* and *Anguillospora crassa*. Key gene families such as cytochrome P450 enzymes, virulence factors, effector proteins, and carbohydrate-active enzymes (CAZymes) were analyzed to understand their roles in temperature and lifestyle adaptations, uncovering possible alternative lifestyle mechanisms. Our findings reveal that Helotiales fungi possess genes associated with nutrient acquisition, pathogenicity, and symbiotic relationships strongly adapted to cold environments that might be impacted by global warming. On the other hand, some species demonstrate potential for adaptation to warmer climates, suggesting increased activity in response to global warming. This study reveals the adaptive mechanisms enabling Helotiales fungi to thrive in both cold and warm environments. These findings provide valuable insights into their ecological success and evolutionary resilience, which may facilitate their ability to transition between pathogenic, symbiotic, and saprobic phases in response to changing environmental conditions.

## 1. Introduction

Helotiales, first described by Nannfeldt in 1932, is an order within the class Leotiomycetes, subdivision Pezizomycotina, and division Ascomycota [1]. It represents the largest order of Ascomycota fungi and the most taxonomically diverse, encompassing over 6000 species distributed across 53 families and 630 genera. Of these, 20 genera have uncertain phylogenetic placement (*incertae sedis*) [2,3,4,5,6]. Helotiales, the most diverse order within Leotiomycetes, exhibits significant diversity both morphologically and phylogenetically and is often regarded as polyphyletic [7]. It encompasses a wide range of teleomorph forms, ranging from small apothecia to more complex structures such as cleistothecia (e.g., in the family *Erysiphaceae*) and perithecia (e.g., in the family *Loramycetaceae*). The majority of Helotiales species possess small apothecia, often measuring less than 2 mm in diameter [8]. They can be either sessile or stipitate, ranging in color from dark to brilliant, and can either appear on the surface or emerge from the plant host [9]. The apothecia have a cup-shaped or disk-like appearance [10].

The teleomorphic species of the Helotiales are characterized by inoperculate asci which reside in discoid, turbinate, or clavate ascocarps ranging in size from the hardly visible members of the *Hyaloscyphaceae* to more prominent members of the *Sclerotiniaceae* [11].

Species in Helotiales thrive in various ecosystems and cover a broad range of niches, having been described as plant pathogens, endophytes, nematode-trapping fungi, mycorrhizae, ectomycorrhizal, parasites, fungal parasites, terrestrial saprobes, aquatic saprobes, root symbionts, and wood rot fungi [8,9]. However, 70% of Helotiales species (known or hypothesized) still have unknown lifestyles (FUNGuild data) [12].

Helotiales, a relatively understudied order of fungi, are gaining recognition for their role in aiding plant nutrients facilitated by fungi [13]. Numerous studies have shown that inoculating plants with Helotiales strains can enhance plant growth through both symbiotic associations and indirect mechanisms elevating nutrient availability by mineralizing and transferring nutrients to their hosts, leading to increased plant biomass and elevated levels of soil nutrients, such as phosphorus and nitrogen [13,14].

Beneficial Helotiales may also mediate plant adaptation in circumpolar-alpine environments [13]. They are among the most representative orders of fungi in those environments, reaching over 41% of the biomass in soils [15,16,17,18,19]. In permafrost soils, members of Helotiales demonstrate a remarkable ability to produce a diverse array of extracellular enzymes at low temperatures. For instance, their cellulase activity allows them to degrade ancient carbon more efficiently than any other taxa [17,19,20]. Similar enzymatic capabilities to perform under temperatures are also found in freshwater fungi to degrade leaf-litter in streams [21], which contributes to a higher abundance of freshwater fungi in colder seasons [22,23,24].

Climatic factors, such as temperature and water availability, significantly influence fungal biomass, diversity, and community composition [25,26,27], reducing enzymatic activity and altering fungal community composition and function, particularly in colder environments [28,29,30]. However, fungal responses to global warming differ depending on their taxa and ecological roles (guilds) [18]. For instance, plant-pathogen fungi may become more abundant under organic farming in drier and warmer climate zones and migrate to historically colder regions due to global warming [27]. While the influence of climate change on phytopathogen evolution remains unclear, previous research has shed light on the impact of increased temperature on their reproduction rate and population density, which can contribute to a faster accumulation of mutations [31,32,33]. Therefore, understanding the fungal genome machinery for adaptation to cold and thermophilic temperatures is fundamental, for example, to warn against the extinction of psychrophilic species or the emergence of thermophilic pathogenic fungi [34].

Thus, the present study seeks to address these gaps via a comprehensive comparative genomic analysis of the Helotiales fungi, with an emphasis on genetic adaptation mechanisms to thrive in cold and warm conditions. In this regard, genes associated with lifestyle adaptation, such as cytochrome P450s, virulence factors, effector genes, and carbohydrate-active enzymes, will be analyzed for their temperature adaptation, providing insights into the fungi’s evolutionary resilience. This analysis may also serve as a foundation for predicting how this group might transit between pathogenic, symbiotic, and saprobic lifestyles in response to environmental changes. The identification of cold-adapted genes provides critical insights into the molecular mechanisms enabling Helotiales to thrive in cold climates. Similarly, the presence of thermo-adapted genes provides a framework for identifying species most vulnerable to global warming, highlighting the need for conservation efforts to protect their biodiversity. Furthermore, the presence of thermo-adapted genes may reveal the potential for certain species to become more active in warming environments, carrying significant implications for ecosystem dynamics. By addressing these aspects, this study aims to support biodiversity conservation strategies, provide a deeper understanding of potential ecosystem impacts, and enhance predictions regarding the broader effects of global warming on fungal communities and their ecological functions.

## 2. Materials and Methods

Cultures of *Gyoerffyella rotula* (HÖHN.) MARVANOVÁ (ex-type strain: CCM F-400) and *Anguillospora crassa* INGOLD (strain: CCM F-13483) were sourced from the Czech Collection of Microorganisms (CCM) and incorporated into the internal collection of the Leibniz Institute DSMZ German Collection of Microorganisms and Cell Cultures under the identifiers DSM 103267 and DSM 105023, respectively. The fungi were cultivated on agar plates containing 2% malt extract (Feelwell GmbH, Gnarrenburg, Germany) with Oxoid brand agar (Germany) at 16 °C in a cooling facility for DNA extraction purposes. To confirm the taxonomy of the strains, Sanger sequencing was performed on the internal transcribed spacer (ITS) and a section of the large subunit gene of the rDNA operon. This was carried out using the primers ITS1F (5′-CTTGGTCATTTAGAGGAAGTAA-3′) [35] and LR5 (5′-TCCTGAGGGAAACTTCG-3′) [36]. Sequencing was performed by Eurofins (Hamburg, Germany). The resulting sequences were manually curated using Sequencher v5.4.6 (http://www.genecodes.com, accessed on 15 November 2023) and subsequently analyzed with BLAST against the National Center for Biotechnology Information (NCBI) nucleotide database (https://www.ncbi.nlm.nih.gov/, accessed on 5 February 2024).

After confirming the taxonomy of the strains, 2 small inocula of fungal biomass from culture plates were transferred to individual 1 L flasks containing potato-glucose liquid medium (Carl Roth, Karlsruhe, Germany) and placed in a cooling room at 16 °C on a shaker (Bottmingen, Switzerland) set to 120 RPM. The samples were kept in motion until sufficient biomass (14 g) was accumulated for genome extraction. Genomic DNA fragments larger than fifteen kilobases were verified using an Agilent Femto Pulse (Agilent, Santa Clara, CA, USA) before being sent to Macrogen in the Netherlands for Pacbio HiFi genome sequencing. The raw sequence data obtained from Macrogen for *G. rotula* and *A. crassa* were assembled using Flye v2.9.2 [37] and evaluated for quality with Busco v5.2.2 [38], employing the ascomycota_odb10 database.

For the comparative genomics of Helotiales, we obtained 127 genomes of Helotiales with 4 genomes of the closely related outgroup taxa of the order Erysiphales (Leotiomycetes) [39]; the genomes of both groups were obtained from the NCBI database. Environmental categories for species included were applied by information retrieved from literature, i.e., ecological studies and taxonomic descriptions. Only genomes with more than 95% completeness were kept for further analysis.

Genome features of size, GC content, number of contigs, and N50 value were determined using the tool assembly-stats (https://github.com/sanger-pathogens/assembly-stats, accessed on 10 February 2024). The tRNA was determined by tRNA tRNAscan-SE v2.0.12 [40] with default parameters for eukaryotic organisms

The genomes of 129 Helotiales (127 from NCBI and 2 newly assembled) and 4 outgroups, were then submitted to ab initio gene prediction with Braker3 v3.03 [41] with parameters “--esmode” and “--fungus”, where the software tool GeneMarker-ES v4.71 [42] was employed to produce hints to train the AUGUSTUS v3.5.0 [43] in predicting the amino acid sequences of each species.

The in silico proteomes obtained above were submitted to BUSCO to identify single-copy-ortholog genes with the ascomycota_odb10 database as a reference. The resulting data were used as input for the BUSCO_phylogenomics tool (https://github.com/jamiemcg/BUSCO_phylogenomics, accessed on 6 April 2024), with the option “--percent_single_copy 75” clustering the single-copy-ortholog genes, performing their alignment, and alignment trimming. The output was then submitted to IQTREE2 v2.2.5 [44] with the options “-bb 1000 -m TEST --seqtype AA” to generate the Helotiales phylogenomic tree.

The amino acid sequences of each strain were used to predict the secreted enzymes using a combination of SignalP 5.0b [45] and Targetp v2.0 [46] to predict whether a provided protein sequence possesses a signal peptide that directs the protein toward secretion. To better understand the molecular mechanisms associated with pathogenicity and substrate degradation from fungi with different lifestyles of the order Helotiales, we performed a comparative analysis of four genomic traits associated with host infection and substrate degradation for the following genes: cytochrome P450 and virulence genes (from DFVF database) using the proteome sequences, and effector genes and carbohydrate degradation enzymes using secretome sequences (CAZy enzymes) [46].

The secreted proteins were then analyzed using EffectorP 3.0 [47] to identify which secreted genes have effector capabilities that can facilitate plant infection by fungi. The secreted genes were also annotated for Carbohydrate-Active enZymes (CAZymes) with run_dbCAN4 (https://github.com/linnabrown/run_dbcan, accessed on 4 November 2023), a standalone tool of the dbCAN3 web server [48], using the tools HMMER v3.3.2 [49], DIAMOND v2.1.8 [50], and dbCAN_sub from the dbCAN3 web server, against the dbCAN3 database v12. The analysis was performed against all six available classes of CAZys: carbohydrate-binding module (CBM), glycoside hydrolases (GHs), polysaccharide lyases (PLs), auxiliary activities (AAs), carbohydrate esterases (CEs), and glycosyl transferases (GTs). Only sequences identified as CAZy enzymes by at least two tools (HMMER, DIAMOND, or dbCAN_sub) were considered for the results. The proteome obtained from the Braker3 pipeline was annotated using Diamond BLASTp for virulence factors against the DFVF (database of fungal virulence factors) [51] against 2058 genes (accessed on 14 June 2024) known to play a role in the infection of plants, herbs, xyloid, animals, vertebrata, invertebrata, and yeast. In addition, the proteome was also blasted against the Fungal cytochrome P450 database (FCPD) [52] containing 23,742 genes. The Diamond BLASTp alignment settings were applied as follows for P450 and DFVF blast: e-value ≤ 1 × 10^−5^, default identity ≥ 40%, coverage ≥ 40%, max-target-seqs = 1 as described in Cheng et al. (2020) [53].

The gene sequences for P450 genes, virulence factor genes, effector genes, and CAZy genes, were submitted to predict cold and thermal adaptabilities through the machine learning tool ThermoProt [54]. This tool applies the support vector machine method (SVN) on calculated amino acid features to predict the thermostability of proteins, discriminating them into cold-adapted and thermo-adapted. The results were then grouped based on the lifestyle of the fungi to understand the mechanisms of adaptation of each fungal lifestyle to temperature.

The statistical analyses were performed in R v4.2.2 (https://www.R-project.org, accessed on 1 September 2023) using Rbase packages, “ggplot2” [55], “dplyr” (https://dplyr.tidyverse.org/, accessed on 16 January 2024), “cowplot” (https://wilkelab.org/cowplot/, accessed on 16 January 2024), “ggpubr” (https://rpkgs.datanovia.com/ggpubr/, accessed on 16 January 2024), and “multcompView” (https://github.com/lselzer/multcompview, accessed on 16 January 2024). Statistical analysis was performed to assess total gene count differences in six gene categories (tRNA genes, amino acid genes, P450 genes, virulence factor genes, effector genes, and secreted CAZy genes) across the following lifestyles: endophyte, freshwater saprobe, marine saprobe, mycorrhiza, phytopathogen, and terrestrial saprobe. To reduce the impact of different numbers of genomes per environment, we calculated the mean for each category within each environment. In sequence, a one-way analysis of variance (ANOVA) was performed to test for significant differences (*p* < 0.05) among environments, followed by Tukey’s Honest Significant Difference (HSD) post hoc test to identify pairwise differences. Statistically distinct groups were represented using significance letters derived from the Tukey test. Boxplots were generated to visualize the data distribution, incorporating jittered data points to display individual variation.

We compared the distribution of four gene categories (P450 genes, virulence factor genes, secreted CAZy genes, and effector genes) under Thermo and Cold conditions within each lifestyle environment for all environments. For that, paired *t*-tests were conducted to assess significant differences between cold- and thermo-adapted genes with *p*-values annotated for each comparison within the environment. The results were represented in boxplots, incorporating jitter plots to represent individual data points and significance levels from *t*-tests. Custom color schemes differentiated the Thermo (light red) and Cold (light blue) conditions. *p* < 0.05 (*) significant difference; *p* < 0.01 (**) strongly significant difference; *p* < 0.001 (***) very strongly significant difference; *p* < 0.0001 (****) extremely significant difference. The analysis was conducted in R v4.2.2 using Rbase packages, “ggplot2”, “dplyr”, “cowplot”, “ggpubr”, “tidyr” (https://tidyr.tidyverse.org, accessed on 16 January 2024), and “lme4” [56].

## 3. Results

The newly assembled genome of the freshwater fungus *Anguillospora crassa* is 55.47 Mbp in size, with 98.4% genome completeness and an N50 value of 32.04 Mbp. Similarly, the genome of *Gyoerffyella rotula* is 44.16 Mbp, with 98.5% genome completeness and an N50 of 23.24 Mbp. Both genomes exhibit genome sizes and completeness levels consistent with other Helotiales genomes available in NCBI and utilized in this study (Figure S1 and Table S1).

The species *Hyaloscypha* sp. PMI_1271 has the current name *Meliniomyces* sp. PMI_1271, (https://mycocosm.jgi.doe.gov/MelPMI1271_1/MelPMI1271_1.home.html, accessed on 10 February 2024) and the species *Hymenoscyphus varicosporioides* has the current name *Tricladium varicosporioides* [57], although the old name was kept in the NCBI database. Assembly quality statistics were obtained from the genomes and are summarized in (Table S1).

**Figure 1:**
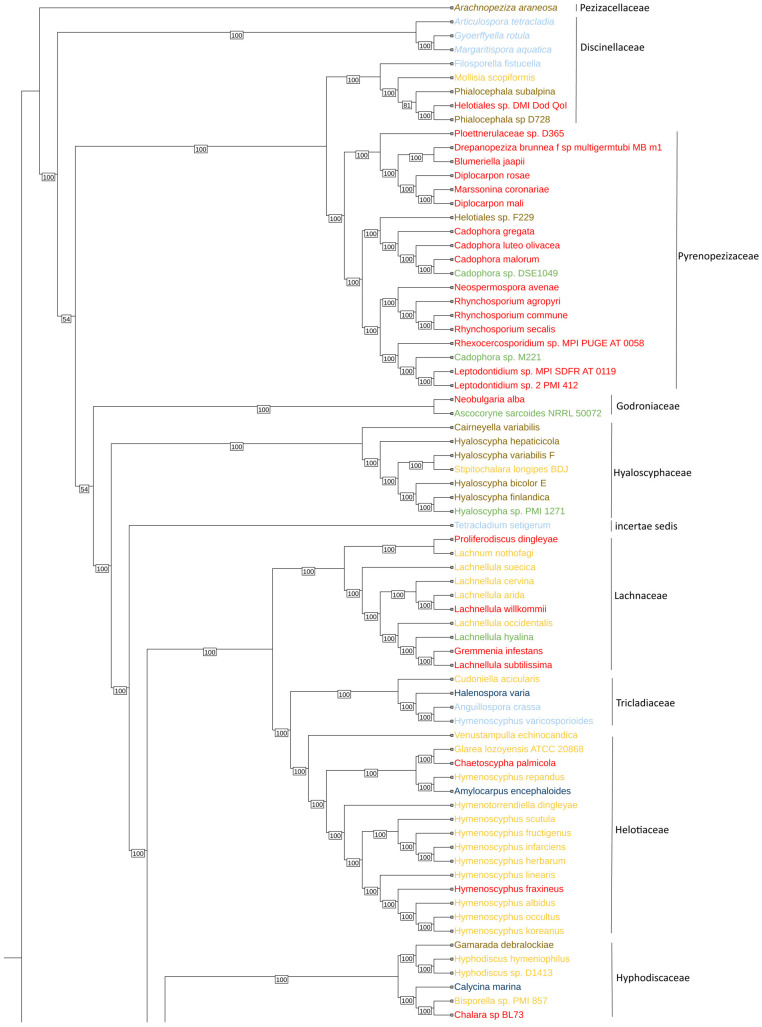

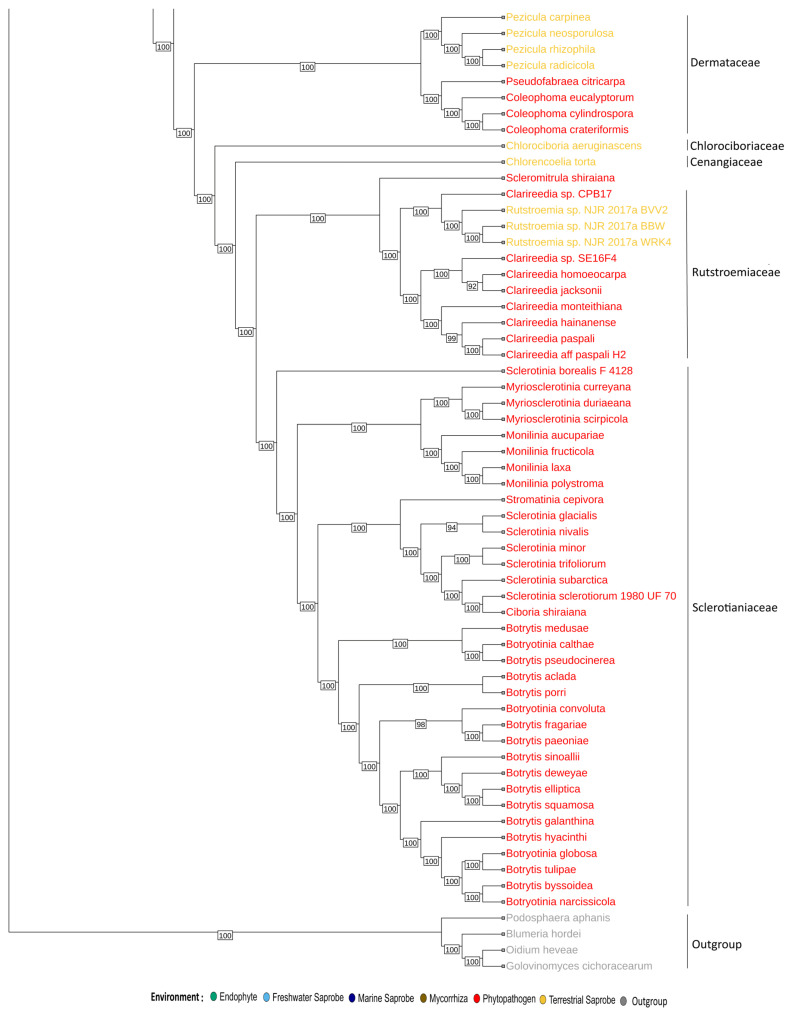
Phylogenomic tree of Helotiales.

The phylogenetic tree (Figure 1) illustrates the evolutionary relationships among diverse fungal species of different lifestyles. The phylogenetic tree was constructed using 1706 single-copy ortholog genes, providing a comprehensive overview of each species’ phylogenetic placement and environmental categorization. The tree was rooted to 4 species of the order Erysiphales from the Leotiomycetes class. The species are categorized into six distinct environmental groups: endophyte (green), freshwater saprobe (light blue), marine saprobe (cyan), mycorrhiza (brown), phytopathogen (red), terrestrial saprobe (yellow), and the outgroup species from Erysiphales (gray).

The concatenation-based ML phylogeny was inferred from 1706 single-copy BUSCO genes found in over 75% of the investigated Helotiales genomes. The tree is rooted in the Erysiphales (NA) order in gray and represents 129 Helotiales species. The species in green are endophytes, in light blue are freshwater saprobes, in dark blue are marine saprobes, in brown are mycorrhiza fungi, in red are phytopathogens, and in yellow are terrestrial saprobe fungi, while in gray are the outgroups. Numbers at clades represent bootstrap values. The log-likelihood of the consensus tree is −39,606,531.707.

The number of tRNA genes in phytopathogens is the highest, with an average of 168.99 followed by the terrestrial saprobe group with an average of 135.06. The species with the largest amounts of tRNA are *Botrytis tulipae* (338), *Pezicula rhizophila* (252), and *Monilinia laxa* (244). The phytopathogens show the highest variability in tRNA gene counts, which might indicate a diverse range of tRNA genes within this group. On the other hand, the mycorrhiza species in this study present had a relatively low number of tRNA genes—*Gamarada debralockiae* (50), *Hyaloscypha hepaticicola* (51), and *Helotiales* sp. F229 (52)—contributing to the low tRNA average observed in the mycorrhiza environment (82.00). The mycorrhiza presents a few outliers, indicating less variability in this group. Phytopathogens, terrestrial saprobes, and mycorrhiza show significant statistical differences, while endophytes, marine saprobes, and freshwater saprobes have intermediate values with averages of 107.80, 113.67, and 136.29, respectively, and no statistical significance with any environment (Figure S2 and Table S2).

The number of amino acids is the highest in mycorrhiza, with an average of 13,911.10, followed closely by terrestrial saprobes with an average of 12,337.09. Mycorrhiza exhibits a high variability in amino acid counts, which may suggest a diverse range of protein-coding potential within this group. Meanwhile, phytopathogens have shown many outliers, indicating greater variability in this group. Two of their species have the second- and third-highest amounts of amino acids: *Leptodontidium* sp. MPI_SDFR_AT_0119 (17,682) and *Leptodontidium* sp. 2_PMI_412 (17,613). On the other hand, phytopathogens also presented the species with lower amounts of tRNA—*Diplocarpon rosae* (7749), *Diplocarpon mali* (7780), *Blumeriella jaapii* (7791), and *Marssonina coronariae* (7808)—contributing to the low amino acid average observed in the phytopathogen environment (10,496.71). The mycorrhiza and phytopathogens are statistically different from the terrestrial saprobe group, whereas endophytes, freshwater saprobes, and marine saprobes have intermediate values with averages of 12,214.80, 12,095.86, and 10,502.67, respectively, and no statistical significance with any environment (Figure S3 and Table S2).

**Figure 2:**
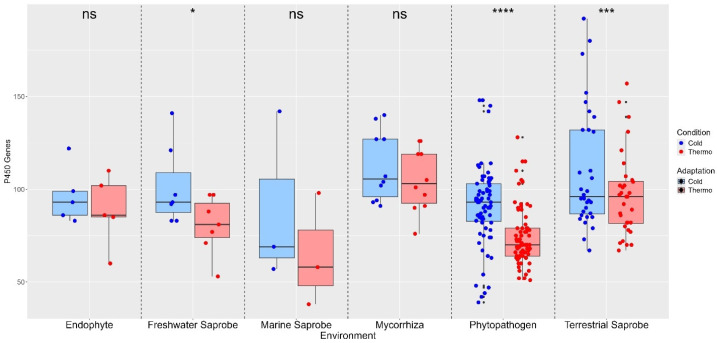
Distribution of P450 gene counts in different environments for cold and thermo adaptation. Statistical comparison between temperature adaptations obtained with the paired *t*-test. Endophyte, marine saprobe, and mycorrhiza presented no significant differences (ns) between cold and thermo conditions. Freshwater saprobe: Significant difference (*) with higher P450 gene counts under cold conditions. Terrestrial saprobe: significant difference (***) with higher P450 gene counts under cold conditions. Phytopathogen: Highly significant difference (****) with cold conditions showing higher P450 gene counts. Where (ns): no significant difference; (*): *p* < 0.05—significant difference; (***): *p* < 0.001—very strongly significant difference; (****): *p* < 0.0001—extremely significant difference.

The P450 gene count varies significantly among the environments, with the highest average count observed in mycorrhiza (464.70 genes) and the lowest in marine saprobes (336.33 genes). Statistical analysis indicates that the mycorrhiza and terrestrial saprobe groups have a significantly higher P450 gene count compared to phytopathogens (333.97 genes), which is the lowest among all environments. The other environments show intermediate average P450 gene counts, with endophytes (402.20), freshwater saprobes (384.33), and marine saprobes (336.33) showing no significant difference in their P450 gene counts when compared to each other and other environments (Figure S4 and Table S2). Regarding temperature adaptations of the P450 gene, there were no significant differences in gene counts between Cold and Thermo conditions in endophytes (ns), marine saprobes (ns), and mycorrhiza fungi (ns). However, significant differences were observed between freshwater saprobes (*), phytopathogens (****), and terrestrial saprobes (***), indicating that temperature adaptation plays a crucial role in the distribution of P450 genes in these environments (Figure 2). Specifically, phytopathogens and terrestrial saprobes show highly significant differences, suggesting a strong adaptive response to cold conditions for P450 genes.

The virulence factor genes across the environments were shown to be relatively close among the groups, ranging from 464.67 in marine saprobes to 527.47 in terrestrial saprobes. Notably, terrestrial saprobes have a significantly higher virulence factor gene count compared to phytopathogens (474.07 genes). In other environments, marine saprobes (464.67 genes), endophytes (507.80 genes), freshwater saprobes (521.86 genes), and mycorrhiza (522.90 genes), do not show significant differences among any environments, indicating a more uniform distribution of virulence factor genes across these environments (Figure S5 and Table S2).

**Figure 3:**
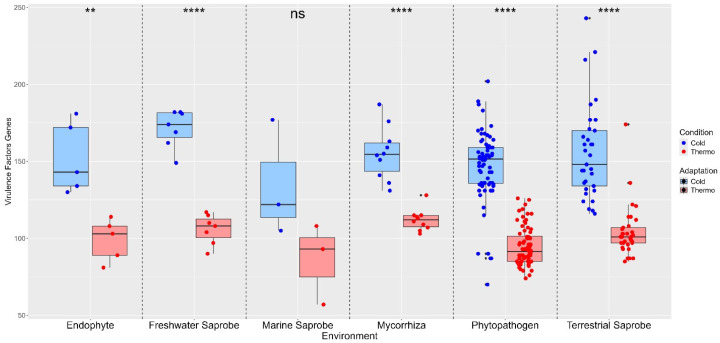
Differences in virulence factor genes between thermo-adapted and cold-adapted conditions across various environments. Statistical comparison between temperature adaptations obtained with the paired *t*-test. No significant difference (ns) between cold- and thermo-adapted virulence factor genes was found in marine saprobes. On the other hand, endophytes (**), freshwater saprobes (****), mycorrhiza (****), phytopathogens (****), and terrestrial saprobes (****) presented significant differences between cold- and thermo-adapted virulence factor genes. Where (ns): no significant difference; (**): *p* < 0.01—strongly significant difference; (****): *p* < 0.0001—extremely significant difference.

In our study, most environmental categories exhibited a higher median count of virulence factor genes for cold adaptations when compared to thermo adaptations, suggesting that these genes are more adapted to cold temperatures across various environments. Marine saprobes are the only lifestyle showing no significant difference in virulence factor gene counts between cold and thermo adaptation (ns). Significant differences were observed in endophytes (**), freshwater saprobes (****), mycorrhiza (****), phytopathogens (****), and terrestrial saprobes (****), indicating that temperature adaptation has a notable impact on the distribution of virulence factor genes in these environments (Figure 3). This pattern suggests that cold-adapted environments may exert selective pressure favoring a higher diversity or abundance of virulence factor genes, enhancing the survival and pathogenic potential of fungi in colder habitats (Figure 3).

The distribution of effector genes among the different environments presents the highest average count found in freshwater saprobes (444.14 genes), while the lowest is in marine saprobes (290.33 genes). Statistical analysis reveals that the effector gene count in phytopathogens (321.67 genes) is statistically significantly lower than in terrestrial saprobes (404.59 genes) but not significantly different from other environments: endophytes (361.60 genes), mycorrhiza (382.60 genes), freshwater saprobes, and marine saprobes. These findings suggest a distinct variation in effector gene counts among the different environmental groups, with freshwater saprobes and terrestrial saprobes having higher counts than other fungal lifestyles (Figure S6 and Table S2).

**Figure 4:**
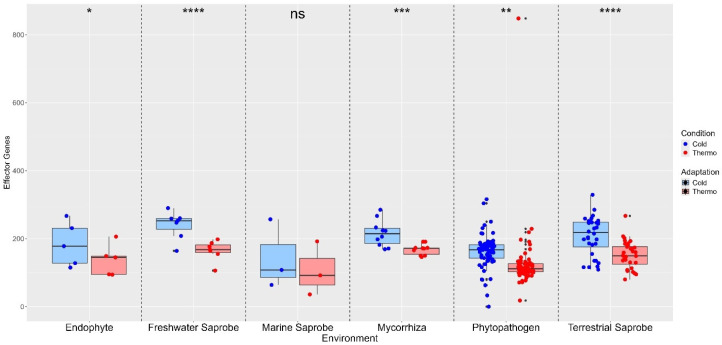
Differences in effector genes between thermo-adapted and cold-adapted conditions across various environments. Statistical comparison between temperature adaptations obtained with the paired *t*-test. No significant difference (ns) between cold- and thermo-adapted effector genes was found in marine saprobes. On the other hand, endophytes (*), freshwater saprobes (****), mycorrhiza (***), phytopathogens (**), and terrestrial saprobes (****) presented significant differences between cold- and thermo-adapted effector genes. Where (ns): no significant difference; (*): *p* < 0.05—significant difference; (**): *p* < 0.01—strongly significant difference; (***): *p* < 0.001—very strongly significant difference; (****): *p* < 0.0001—extremely significant difference.

The effector genes also show higher median counts for cold adaptations than thermo adaptations, indicating that effector genes from these environments may be more adapted to cold temperatures. However, the marine saprobes show no significant difference in effector gene counts between cold and thermo adaptation (ns). Significant differences were found for endophytes (*), phytopathogens (**), mycorrhiza (***), freshwater saprobes (****), and terrestrial saprobes (****), indicating that temperature adaptation plays a crucial role in the distribution of effector genes in these environments. Specifically, freshwater and terrestrial saprobes show highly significant differences, suggesting a strong adaptive response to cold conditions (Figure 4).

The distribution of the secreted CAZy gene counts ranges from 238.00 in marine saprobes to 373.57 in freshwater saprobes. Phytopathogens (263.21 genes) and marine saprobes show significantly lower CAZy gene counts compared to freshwater saprobes, with statistical differences being observed between freshwater saprobes and phytopathogens. The other environments, endophytes (302.60 genes), mycorrhiza (308.70 genes), and terrestrial saprobes (303.91 genes), do not differ significantly from other environments where freshwater saprobes show a notably higher average count (Figure S7 and Table S2).

**Figure 5:**
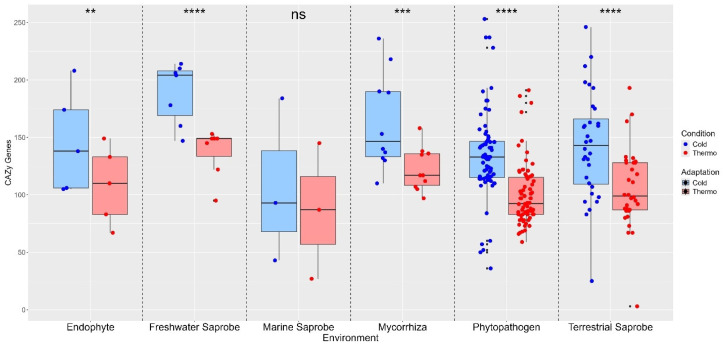
Differences in secreted effector CAZy enzyme between thermo-adapted and cold-adapted conditions across various environments. Statistical comparison between temperature adaptations obtained with the paired *t*-test. No significant difference (ns) between cold- and thermo-adapted CAZy genes was found in marine saprobes. On the other hand, endophytes (**), freshwater saprobes (****), mycorrhiza (***), phytopathogens (****), and terrestrial saprobes (****) presented significant differences between cold- and thermo-adapted effector genes. ns: No significant difference. Where (ns): no significant difference; (**): *p* < 0.01—strongly significant difference; (***): *p* < 0.001—very strongly significant difference; (****): *p* < 0.0001—extremely significant difference.

The secreted CAZy enzymes reveal that, in most environments, cold-adapted organisms exhibit higher median CAZy gene counts compared to thermo-adapted organisms, suggesting that CAZy genes in these environments may be more attuned to cold temperatures. This observation aligns with the patterns seen in effector genes, where marine saprobes showed no significant difference in CAZy gene counts between cold and thermo adaptations (ns). However, significant differences in CAZy gene counts were observed in several other environments: endophytes (**), mycorrhiza (***), freshwater saprobes (****), phytopathogens (****), and terrestrial saprobes (****) (Figure 5).

## 4. Discussion

The phylogenomic tree from 1706 single-copy BUSCO genes generally resembles the topology of the broad sense of the Helotiales concept as shown in Johnston et al. (2019) [2]. In our study, it is used to show the diversity of fungal lifestyles in Helotiales, although *Sclerotiniaceae* and *Pyrenopezizaceae* seem to be inhabited by phytopathogens exclusively. This may be an artifact due to the preferred sequencing of economically important species. Remarkably, *Discinellaceae* seems to be an assemblage of freshwater fungi, as has been shown in earlier studies [2,58,59]. This dataset proves valuable for evolutionary relationships and further taxonomic investigations within the order and provides a starting platform for comparative genomic analysis in this important group of Ascomycetes. One reason for the low support of some clades in our study is the lack of data due to the limited availability of fungal genomes in some Helotiales families. As a result, taxa that are less closely related cluster in our tree. Therefore, we do not draw phylogenetic conclusions from our study.

Global warming refers to the observed heating of the Earth’s land and ocean surfaces; as a result, average temperatures are expected to increase by 2.6 °C higher than before the industrial era by 2046–2065 (https://www.ipcc.ch/sr15/chapter/chapter-3/, accessed on 22 July 2024), accompanied by greenhouse gas emissions and the increased frequency and amplitude of heat waves, as well as altered precipitation patterns. Global warming has emerged as one of the most important issues of our time [60]. Consequently, the potential effect of global warming on a species may depend on its genetic sensitivity to temperature variation [61].

The rise in temperature affects the mechanisms of virulence, epidemiology, reproduction, and survival in fungi [60,62]. With global warming, there is concern that fungal species with pathogenic potential will adapt to higher temperatures, expanding their presence and diseases, which are already being noticed in current elevated temperatures, causing yield losses in crops [63].

On the other hand, for organisms adapted to colder environments, temperature is one of the most important stressors in microorganisms’ survival and ecology [64,65]. For example, psychrophilic fungi possess adaptations that enable them to thrive in cold and nutritionally deficient settings, mostly due to the composition of their cell membranes and proteins [66,67]. Thus, when considering that the majority of the biosphere is permanently cold, which includes the ocean (where 90% of the temperature is below 5 °C) and alpine and polar regions [68,69,70,71,72], the lack of genomic machinery adapted to warmer temperatures may promote the loss of diversity and ecosystem function, whereas enzymes optimized for low temperatures may be less efficient at higher temperatures [61,73].

Cold-adapted freshwater fungi have also demonstrated a lower tolerance for rising temperatures, which may result in shifts in dominance and alterations in species succession patterns, losses in fungal richness and diversity, and impacts on freshwater ecosystem functions, potentially causing biodiversity losses [21,74,75,76,77,78,79].

The fungal species from Helotiales exhibit a varied biogeographical pattern, particularly favoring cold environments with a great prevalence in arctic and alpine environments, with their presence reaching 56% in some studies [13,17]. In the case of the *Sclerotiniaceae* family (Helotiales), structural adaptations in the stroma help the fungi to survive low temperatures [9]. In our study, we observed large amounts of genes adapted to cold temperatures in Helotiales, which suggests a broad range of genes that can be expressed according to adaptations to new niches and lifestyles, as well as new hosts [77], substrates [78], and temperature fluctuations [79].

Our comprehensive gene analysis revealed insights into the functional adaptations of Helotiales fungi in cytochrome P450, virulence factors, effector genes, and CAZy genes, each playing crucial roles in the metabolic processes, pathogenicity, and environmental interactions for cold and thermo adaptations across different environments, revealing significant insights into the functional adaptations and lifestyles of Helotiales fungi.

The cytochrome P450 is a protein family usually referred to as monooxygenases or multifunctional oxidases which can transfer electrons to oxygen and catalyze the oxidation of various organic compounds [80]. They are present in all living organisms and are involved in a wide range of metabolisms of various organic compounds and the biosynthesis of secondary metabolites [34,80,81,82,83] with both endogenous and exogenous properties [84]. In fungi, cytochrome P450 enzymes regulate many different physiological mechanisms, including fertility and fitness [85], playing an important role in environmental adaptation since lifestyle influences P450 composition in the genome [86]. The cytochrome P450 is speculated to play a role in cold tolerance in psychrophilic organisms [87]. As suggested in our results, the distribution of P450 genes may enhance metabolic versatility and detoxification processes in colder environments, showing the importance of these genes in ecological adaptation and niche specialization. The lack of significant differences in endophytes, marine saprobes, and mycorrhiza between cold and thermo conditions indicates a consistent metabolic demand irrespective of temperature variations. However, the limited number of species represented by endophytes (5), freshwater saprobes (7), marine saprobes (3), and mycorrhiza (10) may not reflect the full diversity and adaptive potential of these ecological groups, potentially leading to underestimation of their metabolic or genetic variability in response to temperature adaptations. Other factors, such as genome size and quality, as well as the ecological diversity and representation within the dataset, may introduce biases or disturbances in the results, highlighting the need for more comprehensive genomic data to fully understand their lifestyle adaptations and ecological roles across diverse environments.

Phytopathogenic fungi may have a relatively large number of cytochromes P450 monooxygenases that are involved in fungal pathogenesis due to their ability to neutralize antibiotics produced by the host [85,88,89]. However, our results show low P450 counts in phytopathogens, which might reflect a dynamic set of genes tailored for pathogenicity to specific species-host interaction. Despite that, the presence of outliers within the phytopathogen environment suggests that some species possess an expanded P450 repertoire, potentially reflecting their adaptation to a wide range of host plants and environments (Figure 2), as observed in [90,91,92]. However, there is a limited amount of information available on the relationship between P450 genes and virulence in plant pathogenic fungi [93].

Contrary to phytopathogens, endophyte fungi live within plant tissues without causing disease-related symptoms [91], and their P450 enzymes likely play roles in modulating plant hormone levels, producing antifungal compounds, and degrading plant secondary metabolites [94]. The endophytic lifestyle may require a more diverse set of P450 enzymes than phytopathogens to navigate the complex chemical environment within plant tissues, which offers a protection barrier for the fungi against the biotic factors of the external environment of the host, such as the temperature, and therefore our results show no variation in temperature adaptations for P450.

Cytochrome P450 also impacts on the ecological roles of fungi serving as saprotrophs or decomposers [95]. When P450 is coupled with CAZy enzymes, it also improves the breakdown of compounds at lower temperatures [63,81,96]. For example, in the aquatic fungi *Clavariopsis aquatica*, the P450s are involved in the degradation of leaf-litter when coupled with other enzymes like peroxidases, laccases, cellulose, and hemicellulose [97]. Therefore, our results suggest that the terrestrial and freshwater saprobes likely require a more diverse set of P450 enzymes than marine saprobes to break down a variety of organic materials in their environment, suggesting greater metabolic flexibility, which is crucial for their role in decomposing plant litter and other organic matter. This flexibility may enable them to exploit a wide range of substrates, contributing to their ecological success in their respective ecosystems. They are also involved in the metabolization of pollutants by producing phenoloxidases, peroxidases, low-molecular-weight redox mediators, and intracellular degradation enzymes such as the cytochrome P450 complex [97].

Mycorrhiza exhibited the highest average P450 gene count for cold and thermo adaptations, indicating a robust capacity for metabolic activity, which is critical for competitive interactions within the soil environment and required for symbiotic interactions with plant roots. Our results diverge from what was found by Nsele et al. (2023) [86], where saprotrophs had more P450s than mycorrhizal fungi.

Virulence factors are generally broad in scope and include various molecules that contribute to a pathogen’s ability to cause disease, which may include adhesins, toxins, and enzymes, and may alter the morphological structure of the cell, promoting the development of appressoria, microsclerotia, and conidia, enabling the pathogen to establish itself within the host and enhance its disease-causing potential [51,98,99,100,101]. Therefore, virulence factors may not be necessarily secreted by the fungi to the extracellular environment.

When infecting plants, the fungi produce virulence factors that can be enzymatic or non-enzymatic proteins, secondary metabolites, and small RNAs [96,102]. PAMP-triggered immunity is the result of the plant immune system’s recognition process of inherent molecules of the pathogen, called pathogen-associated molecular patterns (PAMPs), and it is used by plants to initiate a response to cease or ameliorate pathogen colonization [103]. Thus, understanding the mechanism of fungal pathogenicity caused by phytopathogen fungi and their virulence is important for the development of strategies to fight disease with economic importance [97], not only in plants but also in other host organisms. In our findings, phytopathogens have a lower average count of virulence factors compared to saprobe environments, mycorrhiza, and endophytes, which may reflect a specialized set of virulence genes for each species that are highly effective against particular host defenses, supporting the hypothesis that phytopathogens have evolved through a trade-off between broad metabolic capacity and focused pathogenic efficiency (Figure 2).

Mycorrhizal fungi present a high count of virulence factor genes that may modulate host immune responses and facilitate nutrient exchange; virulence factors in mycorrhizal fungi contribute to a mutualistic interaction that benefits both the fungi and the host plant, which is especially observed with Helotiales fungi in alpine and arctic environments [13]. The uniform distribution of virulence genes across mycorrhizal fungi suggests a conserved set of mechanisms that are essential for their symbiotic function. A similar statement can be made about the endophyte fungi virulence factors, as they might be involved in maintaining a stable coexistence within the host plant. In addition, it suggests the potential to switch between mutualistic and pathogenic lifestyles under different environmental conditions [104] (Figure 2).

The terrestrial and freshwater saprobes displayed the highest average count of virulence factor genes, suggesting that these fungi possess a robust arsenal of virulence factors. For example, some aquatic fungi can produce photolytic enzymes, which are among the most important virulence factors produced by fungi pathogenic to fish, crustaceans, and even insects found in the aquatic environment [105]. While these fungi are primarily decomposers, the presence of a high number of virulence genes indicates that terrestrial saprobes may also engage in occasional pathogenic interactions, reflecting their need to interact with a variety of hosts and compete in dynamic environments. Saprobe fungi can become opportunistic pathogens through the development of virulence factors evading the immunologic defense mechanism of the host [106].

In freshwater fungi, the ability to attach to and colonize organic matter is crucial to their ecological role, especially in dynamic environments such as streams and rivers [107]. Virulence factors drive the production of conidia and appressoria—structures central to the successful colonization and survival of fungi in aquatic habitats [57,107]. Conidia, acting as asexual spores, are the primary means of dispersal, enabling fungi to spread through water and come into contact with suitable substrates, such as plant litter or other organic material [108]. Upon reaching the surface of the organic matter, the conidia germinate rapidly, producing germ tubes that develop into appressoria [107,109]. These specialized structures ensure firm attachment to the substrate, allowing the fungus to withstand the often-turbulent conditions of freshwater ecosystems and continue with colonization and nutrient extraction [109,110]. Since appressoria is largely associated with pathogenic fungi [111,112], they could use this trait to switch between an endophytic lifestyle when appropriate. Meanwhile, marine saprobes, with lower virulence factor counts, might be adapted to the more stable and nutrient-rich conditions of marine environments, where the need for a diverse set of virulence factors is reduced (Figure 2).

In addition, the ability to grow and infect the host at different temperatures is largely associated with virulence factors [62,113,114]. Therefore, our study revealed that most environments exhibit higher median counts of virulence factors adapted to cold than to thermo temperatures, with significant differences observed in several environments. Higher median counts of virulence factor genes under cold conditions in freshwater saprobes, endophytes, mycorrhiza, phytopathogens, and terrestrial saprobes suggest an adaptive mechanism enhancing survival and pathogenic potential in cold environments of the Helotiales fungi.

Effector genes are a more specific subset of virulence factors generally characterized by their ability to translocate into host cells and interact with host targets to suppress immune responses or alter cellular processes [115,116]. They play a crucial role in the interactions of fungi with their host plants, especially in the context of phytopathogens, endophytes, and mycorrhizal fungi. They can overcome the first layer of plant immunity (PAMP-triggered immunity) with the help of secreted proteins, which are important for virulence. The second layer involves intracellular host resistance immune proteins that recognize pathogen effectors encoded by avirulence genes (Avr genes) [103,115,116,117,118]. The effector-triggered immunity results in disease resistance and, typically, hypersensitive cell death at the infection site. Successful pathogens can avoid recognition by the plant’s immune system by diversifying or acquiring new effectors promoting disease development [116,117,118,119,120,121]. These genes are involved in manipulating the host’s immune responses, allowing the fungi to colonize and interact with the plant effectively [120].

The ability of these fungi to adapt to different temperatures, particularly warmer ones, is essential for their survival and continued interaction with their hosts. Among the phytopathogens, endophytes, and mycorrhizal species examined, *Diplocarpon rosae* shows a notable decrease in effector gene expression in response to warmer conditions. This decrease suggests that *Diplocarpon rosae* may be less adapted to warmer environments compared to other phytopathogens, endophytes, and mycorrhizal fungi. The reduction in effector gene expression could result in a diminished ability to interact with host plants, possibly leading to a decrease in its pathogenicity or symbiotic efficiency. On the other hand, the phytopathogen *Botrytis byssoidea*, in our study, shows only thermo-adapted genes without any cold-adapted genes, showing very high adaptation to temperature and a potential to increase their activity in global warming events.

Another important mechanism of fungi during infection involves the CAZy enzymes [121]. The phytopathogens use those enzymes to help the evasion of plant immune responses, which enables fungi to survive and propagate in a variety of environments, as well as for the breakdown of the complex polysaccharides found in plant cell walls, which aids in the nutrient acquisition and infection processes [122,123,124,125]. The activity of pathogen enzymes has been directly correlated to the severity of plant disease, which indicates their important role in pathogen colonization. For example, proteases destroy the plant cell walls’ structural proteins, allowing the pathogen to obtain nutrients for its growth and evade plant defenses [103,126,127,128]. Plant defense response is also triggered by the action of those enzymes against the plant’s oligosaccharides [128]. In addition, phytopathogenic and saprophytic fungi can secrete CAZy enzymes into their surroundings to degrade a variety of host-related proteases [129,130]. This degradation mechanism has potential benefits in eliminating the activity of antifungal host proteins and providing nutrients [129,130,131].

Despite helping saprophytic fungi in an alternative lifestyle to overcome plant cell wall defense when infecting the host, the CAZy enzymes in this group are normally associated with the degradation of plant and wood material to break down polysaccharides and obtain carbohydrates (such as starch, cellulose, cellobiose, sucrose, mannose, xylose, maltose, glucose, and galactose), acquiring a large diversity of plant cell wall-degrading or wall-modifying enzymes [21,131]. The CAZymes of classes Carbohydrate Esterase (CE), Glycoside Hydrolase (GH), and Polysaccharide Lyase (PL) are often referred to as plant-litter-degrading enzymes because they play crucial roles in the degradation of plant biomass [122,132].

The higher amount of CAZy genes found in terrestrial saprobes and the significant difference in the abundance of CAZy genes between freshwater saprobes and phytopathogens, as noted in our study and supported by previous research [133,134], highlights the distinct ecological and functional roles of these fungi. Freshwater saprobes are equipped with a diverse array of CAZy enzymes, which are essential for breaking down complex carbohydrates from decaying organic matter, such as plant debris, as a carbon source in the aquatic environment, emphasizing their critical role in the lifestyle adaptation of freshwater saprobes.

Our study demonstrates the crucial role of CAZy enzymes in fungal adaptation to cold environments across various ecological niches. The higher median CAZy gene counts in cold-adapted fungi, particularly in environments such as those inhabited by endophytes, mycorrhiza, freshwater saprobes, phytopathogens, and terrestrial saprobes, suggest that these enzymes are more actively involved in nutrient acquisition and immune evasion at lower temperatures. This pattern indicates a selective advantage for fungi with robust CAZy activity in colder climates. In contrast, the lack of significant difference in CAZy gene counts between cold- and thermo-adapted marine saprobes suggests that temperature may not be the primary factor influencing CAZy expression in marine environments. Instead, other factors like salinity or pressure might play a more significant role.

The CAZy genes are essential for saprobic fungi to thrive in various environments. A critical aspect of their adaptability is how these enzymes function across different temperature ranges, especially under warmer conditions. Based on our data, the terrestrial saprobe *Pezicula carpinea* appears to have the least adaptation to warmer temperatures among the saprobe species examined. The minimal number of CAZy genes expressed under warm conditions (only 4 compared to 26 in cold) suggests that this species may struggle to maintain its saprobic functions as temperatures rise. This could result in a reduced ability to decompose organic materials, potentially affecting their ecological role in warmer environments, and, therefore, *P. carpinea* could be an important species for conservation.

Our results suggest that Helotiales fungi are cold-adapted, but they may metabolize substrates that are not used at lower temperatures when temperature increases [79,134] and perhaps change to an alternative lifestyle when environmental conditions are not favorable. Different lifestyle fungi have been reported to react differently to warming [135]. Terrestrial saprobes, for example, tend to increase their activity with the elevation of temperature [136], while freshwater saprobe seems to be negatively affected in terms of sporulation, leaf processing, and the assemblage of their communities [76,137,138].

Our study shows that Helotiales fungi can have an alternative lifestyle. The presence of CAZy enzymes in phytopathogens, endophytes, and mycorrhiza shows that they can present substrate degradation activities that could go beyond plant infection; in the same way, the saprotrophic lifestyle fungi could also present a phytopathogenic or endophytic lifestyle due to the presence of effector genes and virulence factors. For example, the freshwater saprobes in our study presented a rich amount of virulence factors and effector genes, which indicated a potential for a different lifestyle than the saprophytic one, as found in [21,122]. Many species of freshwater fungi have been found as endophytes or mycorrhizal fungi as an alternative lifestyle [139], including some species of freshwater fungi presented in this study that have been found in a symbiotic relationship with plants such as *Anguillospora crassa* [140], *Filosporella fistucella* [141], and *Tetracladium setigerum* [142]. Likewise, phytopathogenic fungi can have diverse lifestyles, including saprotrophic and symbiotic, in which they use different strategies to interact with their host plants [142].

Despite the lack of studies concerning the warming temperature adaptations of phytopathogenic arctic fungi, several plant pathogenic fungi from Helotiales species have been reported from the glacier ecosystem [143], indicating both their cold-adaptation and that glacial systems act as reservoirs of potential pathogens [144]. As global temperatures rise and glaciers continue to retreat, there is concern that these cold-adapted pathogens may be released into new environments, potentially infecting a wider range of hosts. The warming climate may not only threaten arctic ecosystems but also create conditions conducive to the spread of these fungi, leading to new plant diseases in areas previously unaffected. Understanding how these pathogens might adapt to warmer temperatures is crucial in mitigating potential risks to both local biodiversity and agricultural systems.

## 5. Conclusions

The comprehensive comparative genomic analysis presented in this study provides critical insights into the genomic features in host infection, substrate degradation, and temperature adaptations of Helotiales fungi. By analyzing genomes across different fungi lifestyles, we observed notable differences in the distribution of key genes associated with pathogenicity, nutrient acquisition, and environmental interaction.

Our findings highlight the significance of Cytochrome P450, virulence factors, effector genes, and CAZy enzymes in the adaptation of Helotiales to cold and warm environments. Specifically, many fungi in this order exhibit a strong capacity for cold adaptation, which may explain why they are found in such abundance in arctic and alpine regions. These adaptations are likely driven by evolutionary pressures, enabling the fungi to thrive in extreme conditions by optimizing metabolic pathways for colder temperatures. Conversely, certain species show potential for adaptation to warmer environments, which could become more relevant as global temperatures rise due to climate change.

Importantly, this study reveals the flexible lifestyle strategies of Helotiales fungi. While saprobes typically function as decomposers, some species exhibit the genomic potential for pathogenic and symbiotic interactions, indicating that environmental factors might influence lifestyle shifts. This flexibility allows Helotiales to occupy diverse ecological niches and may have broader implications for their role in ecosystem dynamics, especially in response to global warming. Moreover, their adaptation to global warming has shown to be a multifaceted process that will depend on their genetic makeup, ecological roles, and environmental conditions. Continued research is necessary to fully understand the implications of these changes, particularly for fungi in cold ecosystems, where the effects of global warming may be most pronounced. Understanding these dynamics is essential for predicting future ecological shifts and developing strategies to mitigate the impacts of climate change on fungal biodiversity and ecosystem health.

Our results show a valuable resource for further investigations into Helotiales’ evolution, taxonomy, and environmental adaptations. Understanding the genomic basis of temperature adaptation and its influence on fungal behavior is crucial for predicting the future impacts of climate change on fungal communities, particularly those in colder ecosystems. Nevertheless, in vitro studies remain critical for elucidating the capacity of fungi to alter their lifestyles in response to warming, offering deeper insights into their adaptive strategies and ecological flexibility.

## Data Availability

Data are contained within the article and Supplementary Materials. Tables S1 and S2 with the number of features annotated are available at https://doi.org/10.5281/zenodo.14050745 (accessed on 16 August 2024), and the genome sequences used in this research have been deposited in NCBI’s GenBank (their accession numbers can be found in Table S1).

## Supplementary Materials

The following supporting information can be downloaded at: https://www.mdpi.com/article/10.3390/jof10120869/s1. Figure S1: Boxplot showing genome size distribution across fungal environmental categories; Figure S2: Comparative Distribution of tRNA Across Different Environments; Figure S3: Comparative Distribution of Amino acids Across Different Environments; Figure S4: Comparative Distribution of P450 Genes Across Different Environments; Figure S5: Comparative Distribution of Virulence Factor Genes Across Different Environments; Figure S6: Comparative Distribution of Effector Genes Across Different Environments; Figure S7: Comparative Distribution of CAZy Genes Across Different Environments; Table S1: Genome assembly size and quality for every species in this study; Table S2: Genome annotation counts for P450, virulence factors, effectors, and CAZy genes and their temperature adaptation.
