# Supplementary material for: Comparative Genomics of Different Lifestyle Fungi in Helotiales (Leotiomycetes) Reveals Temperature and Ecosystem Adaptations
Source: J Fungi (Basel). 2024 Dec 14;10(12):869. doi: 10.3390/jof10120869 (PMC11678538; doi:10.3390/jof10120869)
Supplement: Supplementary file 1 [file jof-10-00869-s001.zip › SUPP_files_Final figures.pdf]

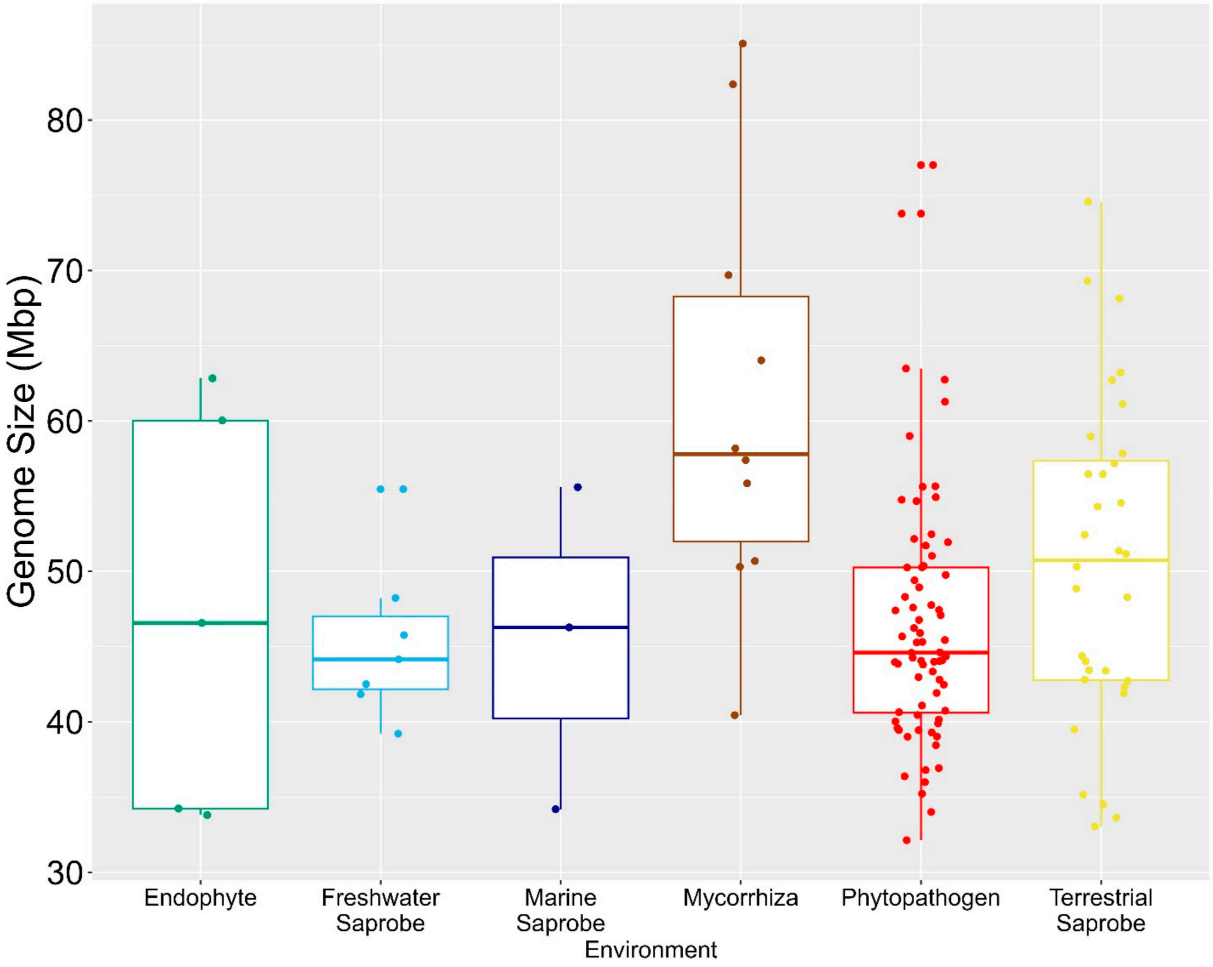


**Figure S1.** Boxplot showing genome size distribution across fungal environmental categories.
Genome size distribution across fungal lifestyles. Box colors correspond to different fungal lifestyles: green (Endophyte), light blue (Freshwater Saprobe), dark blue (Marine Saprobe), brown (Mycorrhiza), red (Phytopathogen), and yellow (Terrestrial Saprobe). Each box represents the interquartile range (IQR) of genome sizes, with the horizontal line inside the box indicating the median. Whiskers extend to the smallest and largest values within 1.5 times the IQR. Individual data points represent genome sizes for specific fungi within each category. The x-axis indicates fungal lifestyle categories, and the y-axis represents genome size.

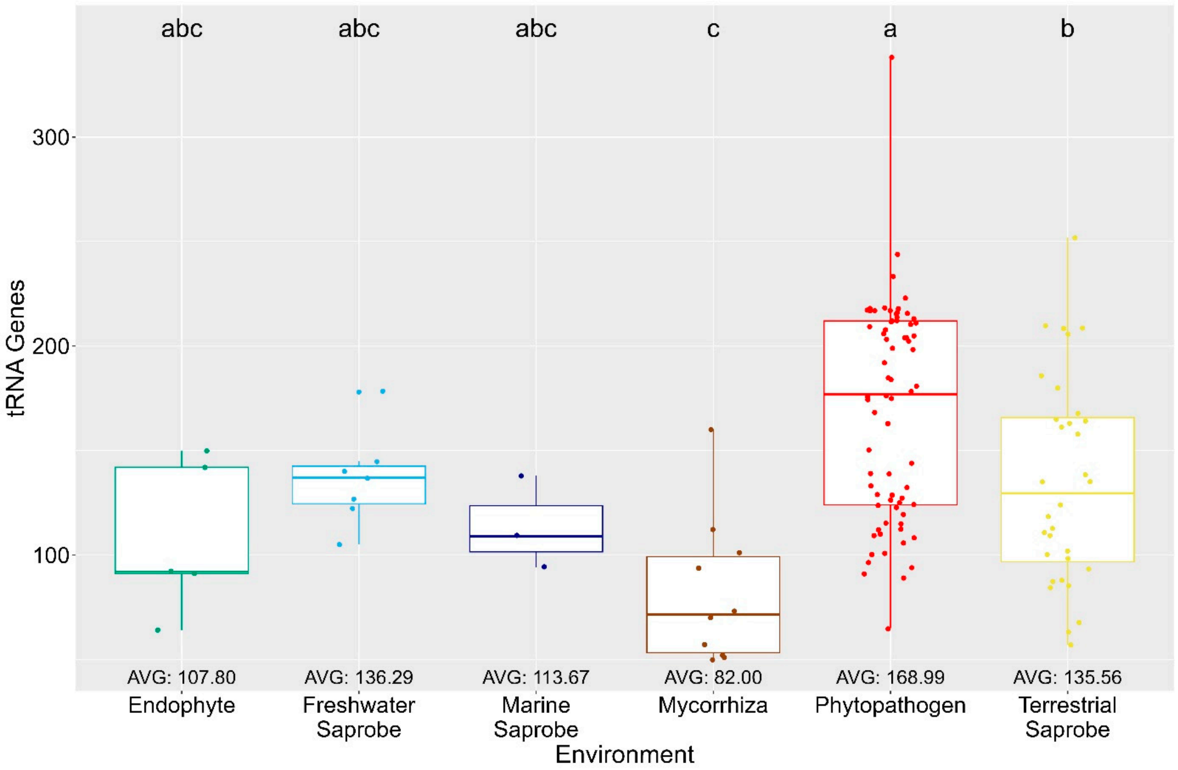


**Figure S2.** Comparative Distribution of tRNA Across Different Environments.

The distribution of tRNA gene counts highlights significant differences among environmental categories, with phytopathogens showing the highest average count (AVG: 168.99) and mycorrhiza the lowest (AVG: 82.00). Significant differences are denoted by different letters (a, b, c). Categories sharing the same letter do not differ significantly. For the statistical analysis, it was implemented ANOVA (Analysis of Variance), followed by Tukey's Honest Significant Difference (TukeyHSD) test for post-hoc comparisons. Color coding: endophyte (green), freshwater saprobes (light blue), marine saprobes (dark blue), mycorrhiza (brown), phytopathogen (red), terrestrial saprobes (yellow).

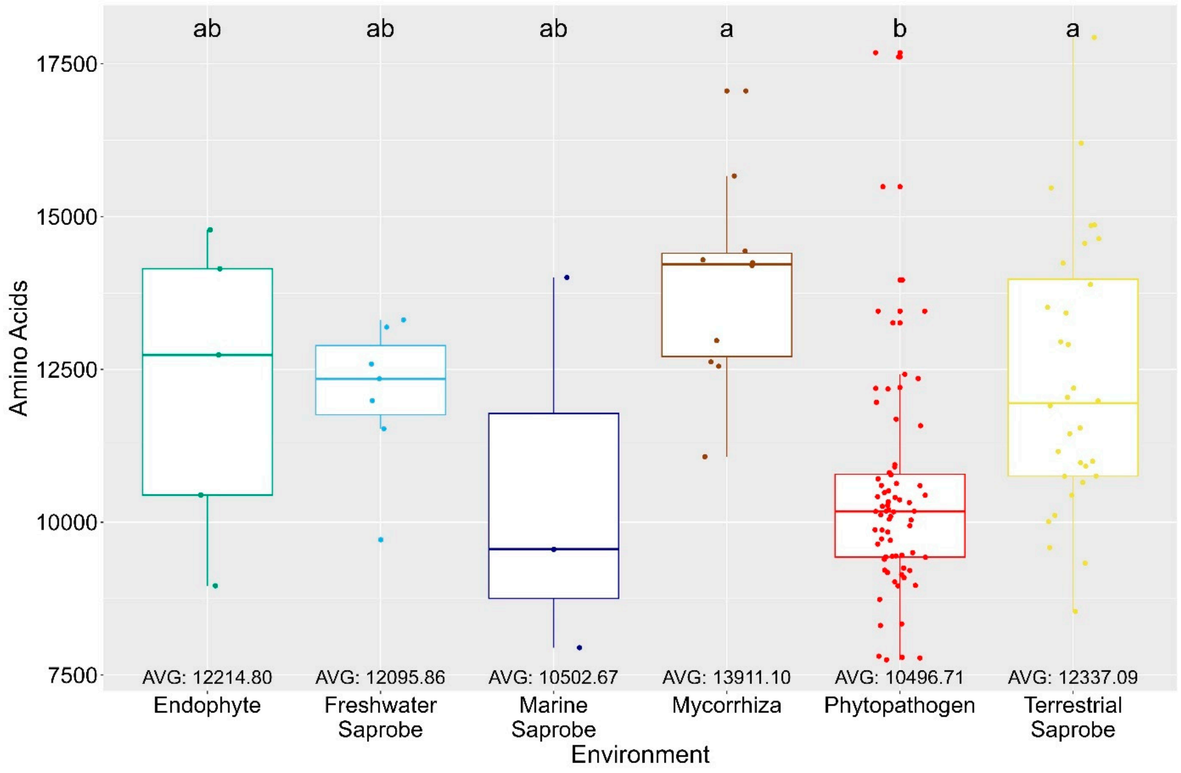


**Figure S3.** Comparative Distribution of Amino Acids Across Different Environments.

The distribution of amino acid gene counts highlights significant differences among environmental categories, with mycorrhiza showing the highest average count (AVG: 13911.10) and phytopathogens the lowest (AVG: 10496.71). Significant differences are denoted by different letters (a, b). Categories sharing the same letter do not differ significantly. For the statistical analysis, it was implemented ANOVA (Analysis of Variance), followed by Tukey's Honest Significant Difference (TukeyHSD) test for post-hoc comparisons. Color coding: endophyte (green), freshwater saprobes (light blue), marine saprobes (dark blue), mycorrhiza (brown), phytopathogen (red), terrestrial saprobes (yellow).

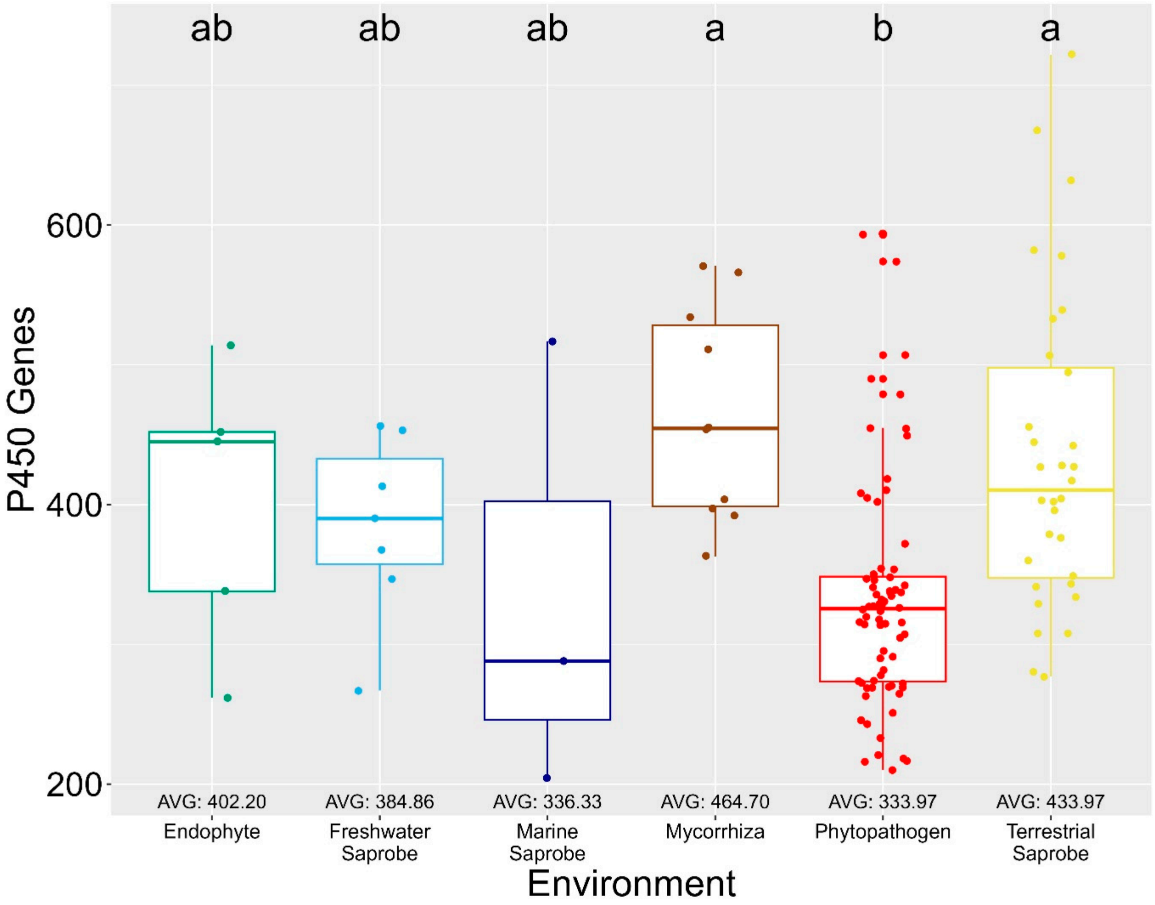


**Figure S4.** Comparative Distribution of P450 Genes Across Different Environments.

The distribution of P450 gene counts highlights significant differences among environmental categories, with mycorrhiza showing the highest average count (AVG: 464.70) and marine saprobes the lowest (AVG: 336.33). Significant differences are denoted by different letters (a, b). Categories sharing the same letter do not differ significantly. For the statistical analysis, it was implemented ANOVA (Analysis of Variance), followed by Tukey's Honest Significant Difference (TukeyHSD) test for post-hoc comparisons. Color coding: endophyte (green), freshwater saprobes (light blue), marine saprobes (dark blue), mycorrhiza (brown), phytopathogen (red), terrestrial saprobes (yellow).

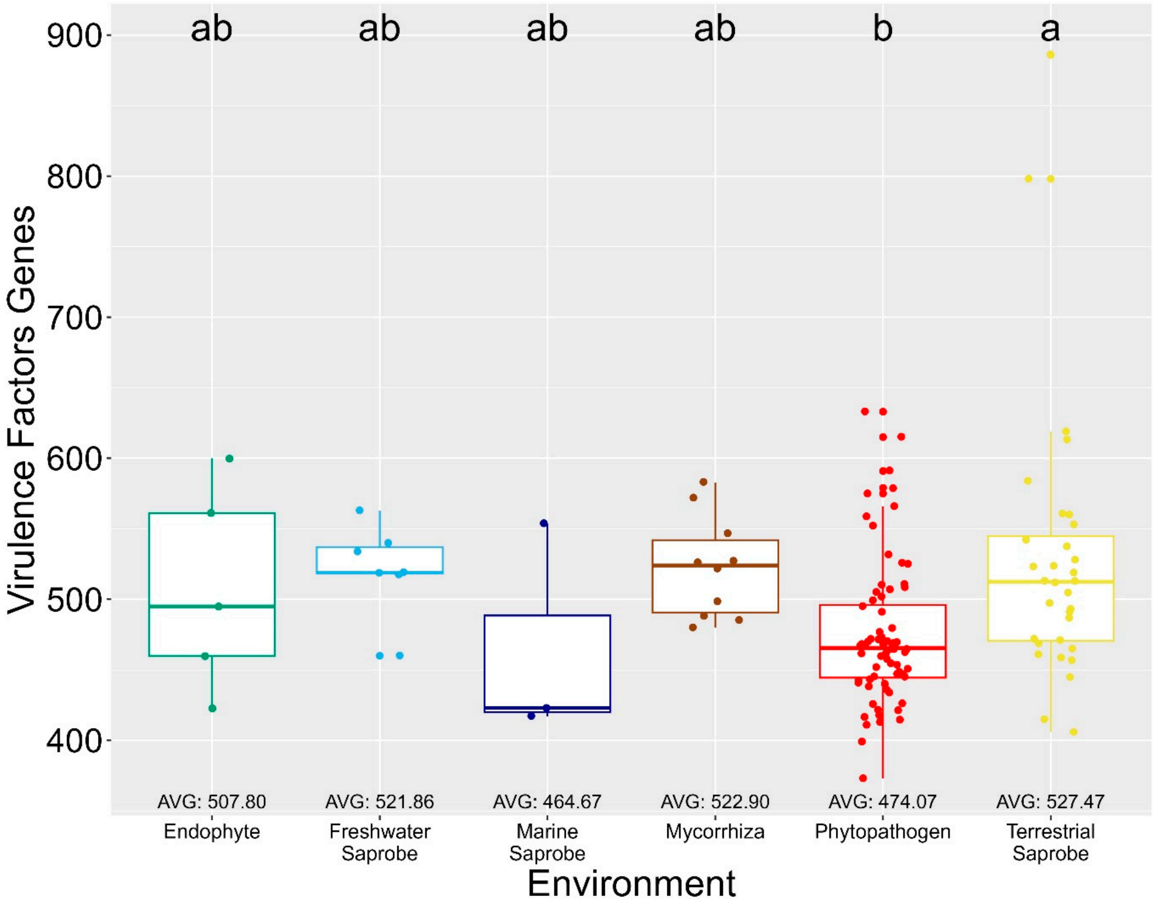


**Figure S5.** Comparative Distribution of Virulence Factor Genes Across Different Environments.
Virulence factor gene counts exhibit a relatively uniform distribution, with terrestrial saprobes having the highest average count (AVG: 527.47) and marine saprobes the lowest (AVG: 464.67). Significant differences are denoted by different letters (a, b). Categories sharing the same letter do not differ significantly. For the statistical analysis, it was implemented ANOVA (Analysis of Variance), followed by Tukey's Honest Significant Difference (TukeyHSD) test for post-hoc comparisons. Color coding: endophyte (green), freshwater saprobes (light blue), marine saprobes (dark blue), mycorrhiza (brown), phytopathogen (red), terrestrial saprobes (yellow).

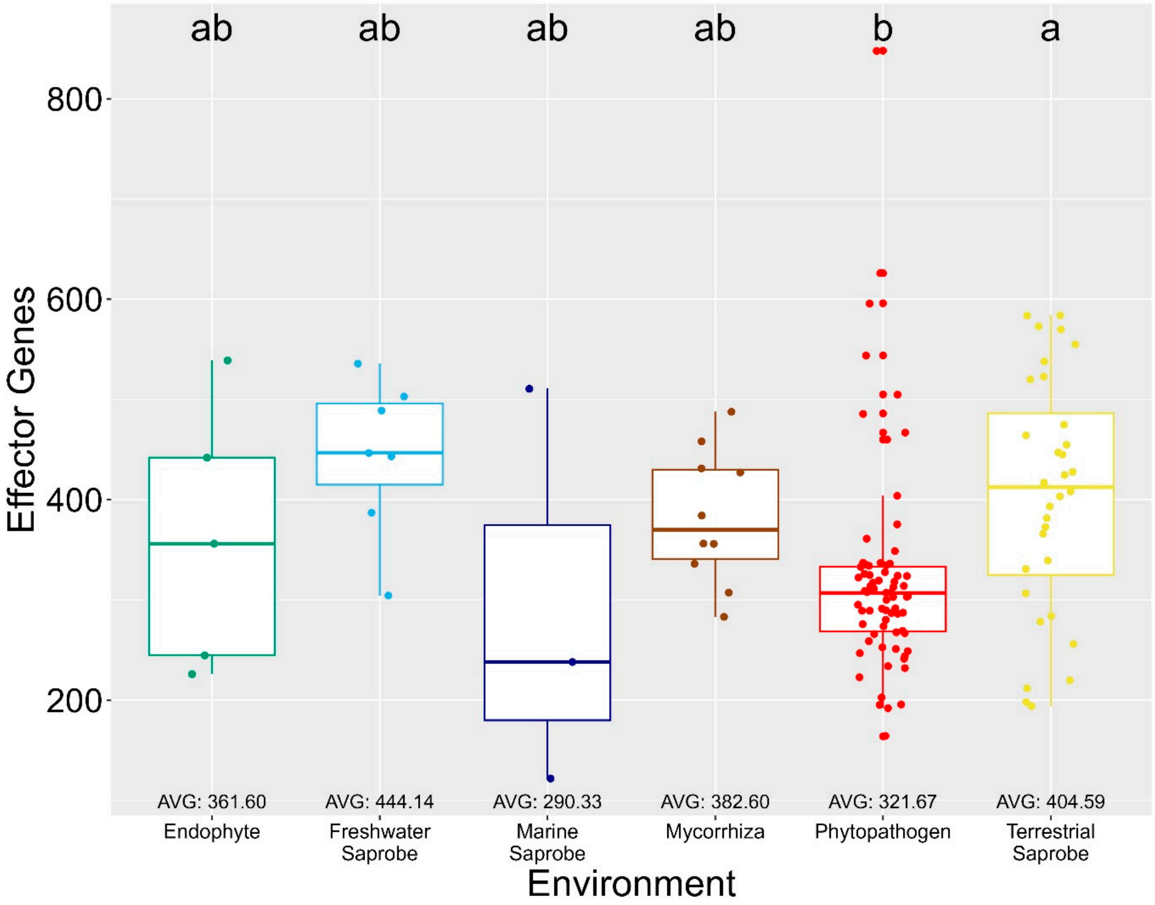


**Figure S6.** Comparative Distribution of Effector Genes Across Different Environments.
The distribution of effector gene counts shows notable variation, with freshwater saprobes having the highest average (AVG: 444.14) and marine saprobes the lowest (AVG: 290.33). Significant differences are denoted by different letters (a, b). Categories sharing the same letter do not differ significantly. For the statistical analysis, it was implemented ANOVA (Analysis of Variance), followed by Tukey's Honest Significant Difference (TukeyHSD) test for post-hoc comparisons. Color coding: endophyte (green), freshwater saprobes (light blue), marine saprobes (dark blue), mycorrhiza (brown), phytopathogen (red), terrestrial saprobes (yellow).

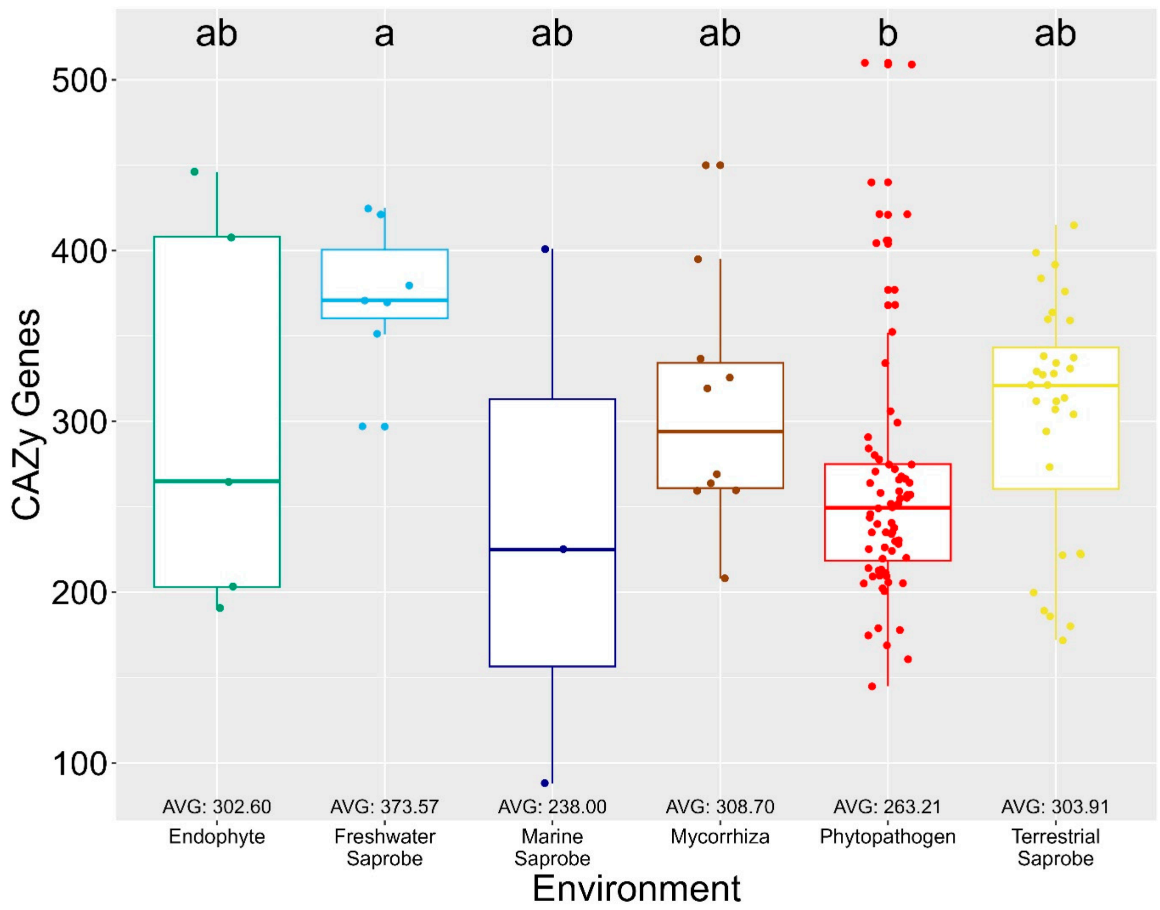


**Figure S7.** Comparative Distribution of CAZy Genes Across Different Environments.
The CAZy gene counts vary across environments, with freshwater saprobes having the highest average (AVG:373.57) and marine saprobes the lowest (AVG: 238.00). Significant differences are denoted by different letters (a, b). Categories sharing the same letter do not differ significantly. For the statistical analysis, it was implemented ANOVA (Analysis of Variance), followed by Tukey's Honest Significant Difference (TukeyHSD) test for post-hoc comparisons. Color coding: endophyte (green), freshwater saprobes (light blue), marine saprobes (dark blue), mycorrhiza (brown), phytopathogen (red), terrestrial saprobes (yellow).
